# Clinical outcomes and patterns of care in the treatment of carcinosarcoma of the breast

**DOI:** 10.1002/cam4.1942

**Published:** 2019-03-12

**Authors:** William R. Kennedy, Prashant Gabani, Sahaja Acharya, Maria A. Thomas, Imran Zoberi

**Affiliations:** ^1^ Department of Radiation Oncology Washington University School of Medicine Saint Louis Missouri; ^2^ Department of Radiation Oncology St. Jude Children's Research Hospital Memphis Tennessee

**Keywords:** breast, breast cancer, carcinosarcoma, chemotherapy, national cancer database, radiation therapy

## Abstract

**Purpose:**

Carcinosarcoma of the breast is a rare yet highly aggressive tumor accounting for <1% of all breast cancers, for which guidance on optimal management and prognosis are sparse. The purpose of this study was to investigate population‐based treatment patterns and overall survival (OS) outcomes in patients with this diagnosis.

**Materials and Methods:**

We queried the National Cancer Database for patients diagnosed with carcinosarcoma of the breast. All patients included were treated with surgery in the form of mastectomy or lumpectomy, with or without chemotherapy and/or radiation therapy. Patients with metastatic disease were excluded. Kaplan‐Meier analysis was used to estimate OS. Univariate and multivariable Cox analyses were used to determine predictive factors of OS.

**Results:**

A total of 329 patients from 2004 to 2012 were identified. Median age at diagnosis was 58 years (range, 24‐90). Patients had T1 (21%), T2 (44%), T3 (25%), or T4 disease (10%). Most patients were node‐negative at diagnosis (77%). Breast conservation surgery was utilized in 33% of patients. Chemotherapy was used in 66% of patients. Less than half (44%) of patients received radiation therapy to a median dose of 50.4 Gy (range 35‐56 Gy), with a median 10 Gy boost used in 76%. With a median follow‐up of 40.0 months, 3‐ and 5‐year OS for all patients was 74% and 60%, respectively. Kaplan‐Meier estimates revealed the 3‐yr OS was 80% in patients receiving chemotherapy vs 59% without chemotherapy (*P* < 0.001). The 3‐yr OS was 82% in patients receiving RT vs 66% without RT (*P* = 0.001). On multivariable analysis, OS was significantly influenced by Charlson‐Deyo comorbidity index, insurance status, clinical T stage, surgical margin status, and treatment group, with trimodality therapy (HR: 0.45, 95% CI: 0.27‐0.78; *P* = 0.004) and surgery plus CT (HR: 0.54, 95% CI: 0.33‐0.90; *P* = 0.02) being associated with the greatest OS. Logistic regression revealed only younger patients were more likely to receive trimodality therapy.

**Conclusions:**

Carcinosarcoma of the breast is associated with relatively poor rates of OS. The addition of CT and RT to surgery improves OS. Trimodality therapy and surgery plus CT were associated with the greatest OS compared to surgery alone.

## 1 INTRODUCTION

Carcinosarcoma of the breast is a rare and highly aggressive tumor which accounts for <1% of all new cases of breast cancer annually.^1^ Histologically, carcinosarcomas are poorly differentiated cancers exhibiting carcinoma cells intermixed with a malignant nonepithelial mesenchymal component that lacks a transition zone between these two malignant cell types.^2^, ^3^ The malignant mesenchymal component in these tumors can include elements of chondroid, osseous, rhabdomyoid and even neuroglial differentiation. Also known as metaplastic breast cancer with mesenchymal differentiation, it is one of the five distinct subtypes of metaplastic breast cancer (MBC) characterized by the 2011 World Health Organization Working Group.^4^ Other subtypes of MBC include squamous cell carcinoma, low‐grade adenosquamous carcinoma, spindle cell carcinoma, and fibromatosis‐like metaplastic carcinoma. These cancers are typically hormone receptor negative, but hormone receptor status does not appear to affect prognosis in these patients.^5^

Compared to invasive ductal (IDC) and invasive lobular (ILC) breast cancers, carcinosarcoma is more likely to present with a larger primary tumor and less likely to have lymphatic involvement at diagnosis.^6^ Carcinosarcoma has an increased tendency for hematogenous dissemination, with lung and pleural metastases being the most common site of distant disease.^7^ Accordingly, MBC typically presents with a more advanced stage at diagnosis and both local and distant failure rates are higher relative to IDC or ILC.^8^ Local recurrence rates are particularly high, with one study reporting 53% at 2 years compared to an expected 10% or less in other invasive cancers, warranting the need for aggressive local therapy.^9^ This more aggressive clinical course for MBC as a whole appears to hold true when compared specifically to triple‐negative breast cancer (TNBC).^10^

As carcinosarcoma is an infrequent entity lacking randomized trials, there is little guidance on the optimal treatment of these tumors. Furthermore, many studies examining carcinosarcoma also include patients with several other MBC subtypes and a heterogeneous array of clinical factors, making accurate prognostication and risk stratification difficult to ascertain. Studies specific to carcinosarcoma treatment and outcomes are limited to only a handful of cases, underscoring the need for further investigation to better understand the clinical course and ideal management of these patients.

Our aim was to evaluate patient characteristics and treatment approaches as they relate to survival outcomes in a modern cohort of patients diagnosed with carcinosarcoma of the breast using a large hospital‐based cancer registry, the National Cancer Database (NCDB). We hypothesized that survival would be improved in patients treated with multimodal therapy with chemotherapy (CT), radiation therapy (RT), or both compared to surgical resection alone.

## 2 MATERIALS AND METHODS

### 2.1 Data source and study population

We reviewed the NCDB Participant User File (PUF) of breast tumors to identify all patients diagnosed with carcinosarcoma of the breast (histology 8980). NCDB is a joint program of the American College of Surgeons and the American Cancer Society. Data are available on patients diagnosed at Commission on Cancer (CoC) accredited cancer centers, capturing approximately 70% of all newly diagnosed malignancies in the United States annually.^11^ Data on patient, tumor, and treatment characteristics are collected and submitted to the NCDB from CoC‐accredited oncology registries using standardized coding and data item definitions. The Health Insurance Portability and Accountability (HIPAA)‐compliant NCDB PUF contains only de‐identified patient and center information and did not require institutional review board approval.

De‐identified data for patients diagnosed with carcinosarcoma of the breast (International Classification of Disease for Oncology, 3rd edition [ICD‐O‐3] code 8980) who were aged 18‐90 and diagnosed between 2004 and 2012 were evaluated. Other non‐IDC/ILC histologies including metaplastic carcinoma NOS, squamous cell carcinoma, and spindle cell carcinoma were excluded. In our analysis, we excluded patients with distant metastatic disease at time of diagnosis and incomplete treatment data. Demographic and clinical data included age, gender, race, year of diagnosis, stage, grade, Charlson‐Deyo comorbidity index (CDCI), treatment location and facility type, primary insurance status, type of treatment (surgery, RT, or CT). Surgery included either mastectomy or partial mastectomy. The groups that were included in our analysis were surgery alone, surgery plus CT, surgery plus RT, and trimodality therapy consisting of surgery plus chemoradiotherapy (CRT).

### 2.2 Statistical analysis

Overall survival was calculated from diagnosis until death or last follow‐up. The Kaplan‐Meier method was used to estimate OS probabilities and Cox analyses were performed. Patients with incomplete data regarding surgery, chemotherapy, or radiotherapy were excluded from survival analysis. In addition to these analyses, age, year of diagnosis, race, insurance status (private vs uninsured vs government insurance), CDCI, T and N stage, facility type (academic vs non‐academic), margin status, surgery type, and treatment strategy were used on univariate analysis. Variables at *P* < 0.05 on univariate testing were entered into multivariable analyses using the Cox proportional hazards model. To confirm appropriate selection of predictive variables entered into multivariable analysis, backwards stepwise regression was utilized. Significance was considered at *P* < 0.05, and all significance levels were 2‐sided. Binary logistic regression was performed to obtain odds ratios for factors predictive of receiving trimodality therapy, with factors with *P* < 0.10 on univariate analysis entered into the model for stepwise selection. IBM® SPSS® Statistics, version 23 was applied for all statistical analyses.

## 3 RESULTS

### 3.1 Demographics, patient, tumor, and treatment characteristics

We identified a total of 329 patients treated between 2004 and 2012. Median follow‐up was 40 months (range 0‐123.8 months). Patient characteristics and treatment strategies are summarized in Table 1. The majority of disease was hormone receptor negative and human epidermal growth factor receptor (HER)2/neu (c‐erbB2) non‐amplified. Surgery was mastectomy in 65.4% and partial mastectomy in 31.6% of patients. Radiation therapy was administered to 43.2% of all patients. Of patients receiving breast conservation surgery, adjuvant radiation therapy was utilized in 71% of patients receiving partial mastectomy. Of patients receiving mastectomy, postmastectomy RT (PMRT) was utilized in 32% of patients. Patients receiving PMRT had pathologic T3 or 4 disease in 63%, pathologic node positivity in 35%, positive margins in 9%, with no patients having at least 1 of the above factors. The median RT dose was 50.4 Gy (range, 35.0‐55.8 Gy). A boost was used in 28.9% of patients, with a median dose of 10.0 Gy (1.6‐18.4 Gy). Chemotherapy was also used as part of initial treatment in 66.0% of patients, of which 18% received neoadjuvant systemic therapy starting at least 30 days prior to definitive surgery and the remaining 82% received adjuvant chemotherapy. Distribution of treatment groups were surgery alone (77 patients, 23.4%), surgery plus CT (96 patients, 29.2%), surgery plus RT (26 patients, 7.9%), and trimodality therapy (116 patients, 35.3%). There were 14 patients (4.3%) with incomplete details regarding either surgery, RT or CT.

**Table 1:** Patient‐ and treatment‐related characteristics (n = 329)

Characteristic	Value
Age, median (range)	58 y (range, 24‐90)
Race	
White	262 (79.6%)
Black or Other	67 (20.4%)
Year of diagnosis	
2004‐2008	176 (53.5%)
2009‐2012	153 (46.5%)
Charlson‐Deyo comorbidity index	
0	267 (81.2%)
1	49 (14.9%)
>1	13 (4.0%)
Insurance status	
Private	156 (47.4%)
Government	151 (45.9%)
Uninsured	16 (4.9%)
Unknown	6 (1.8%)
Setting	
Metropolitan	274 (83.3%)
Rural	42 (12.8%)
Urban	6 (1.8%)
Unknown	7 (2.1%)
Facility type	
Academic	73 (22.2%)
Non‐Academic	223 (67.8%)
Unknown	33 (10.0%)
Clinical T stage	
T1	75 (20.6%)
T2	159 (43.7%)
T3	90 (24.7%)
T4	40 (11.0%)
Clinical N stage	
N0	282 (77.5%)
N1	64 (17.6%)
N2	15 (4.1%)
N3	3 (0.8%)
Surgery	
Mastectomy	215 (65.4%)
Partial Mastectomy	104 (31.6%)
Unknown	10 (3.0%)
Surgical margins	
Negative	290 (88.1%)
Positive	25 (7.6%)
Unknown	14 (4.3%)
Chemotherapy	
Neoadjuvant	40 (12.2%)
Adjuvant	177 (53.8%)
No chemotherapy	112 (44.0%)
Hormonal therapy	
Yes	15 (4.8%)
No	314 (95.2%)
Estrogen receptor status	
Positive	41 (12.5%)
Negative	260 (79.0%)
Not recorded	28 (8.5%)
Progesterone receptor status	
Positive	35 (10.6%)
Negative	265 (80.6%)
Not recorded	29 (8.8%)
HER2/CerbB‐2 status	
Amplified	4 (1.2%)
Non‐amplified	113 (34.3%)
Not recorded	212 (64.5%)
Treatment group	
Surgery alone	77 (23.4%)
Surgery + CT	96 (29.2%)
Surgery + RT	26 (7.9%)
Surgery + CT + RT	116 (35.3%)
Either RT or CT details unknown	14 (4.3%)
Radiation Dose, median (range)	50.4 Gy (35.0‐55.8 Gy)
Radiation Boost	
Yes	95 (28.9%)
No	30 (9.1%)
Either no RT or unknown	204 (62.0%)
Boost Dose, median (range)	10.0 Gy (1.6‐18.4 Gy)

### 3.2 Outcomes

**Figure 1:**
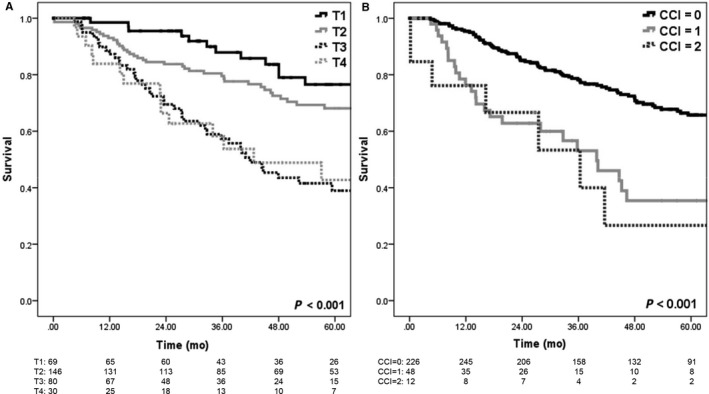
Overall survival. A, by tumor stage (T2 vs T3 and T4 *P* < 0.001). B, by CDCI (between all groups *P* < 0.001)

**Figure 2:**
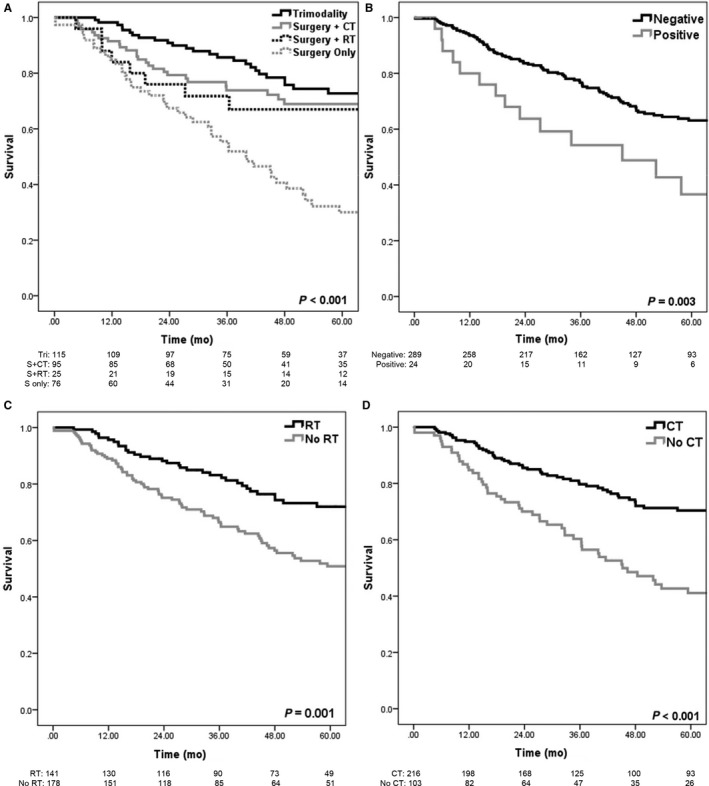
Overall survival. A, by treatment group (*P* < 0.001). B, by surgical margin status (*P* = 0.003). C, by use of RT (*P* = 0.001) and D, use of CT (*P* < 0.001)

The median OS for the entire cohort was 8.7 years. At the time of analysis, 117 patients had died. Estimated 3‐year and 5‐year OS was 74% and 60%, respectively, for all patients. Median OS in patients with clinical T2, T3, and T4 disease was 8.7 (95% CI: 6.8‐10.7), 3.5 (95% CI: 2.6‐4.4), and 3.6 years (95% CI: 1.1‐6.0), respectively (*P* < 0.001) (Figure 1A). There was no statistical difference in OS between patients with clinically node‐negative and node‐positive disease at diagnosis (*P* = 0.166). For patients diagnosed in 2004‐2008 vs 2009‐2012, there was no significant different in OS (*P* = 0.682). Median OS in patients with CDCI of 1 and 2 was 3.3 (95% CI: 2.2‐4.5) and 3.0 years (95% CI: 1.0‐5.1), respectively, while median OS was not reached in patients with CDCI of 0 (*P* < 0.001) (Figure 1B). When stratified by treatment group, estimated 3‐ and 5‐year OS was 85% and 72%, respectively, for patients treated with trimodality therapy, 74% and 68% for patients receiving surgery and CT (but not RT), 67% and 59% for patients receiving surgery and RT (but not CT), and 54% and 30% for patients receiving surgery alone (*P* < 0.001) (Figure 2A). Kaplan‐Meier OS curves of patients stratified by surgical margin status are shown in Figure 2B, with 3‐ and 5‐year OS 76% and 63% for negative margins, and 53% and 35% for positive margins, respectively (*P* < 0.001). When stratified by treatment with chemotherapy, the 3‐ and 5‐year OS was 80% and 70% in patients receiving chemotherapy vs 59% and 41% without chemotherapy (*P* < 0.001) (Figure 2C). The 3‐ and 5‐year OS was 82% and 72% in patients receiving RT vs 66% and 50% without RT (*P* = 0.001) (Figure 2D).

### 3.3 Univariate and multivariable analyses

On univariate and multivariable analysis, CDCI, T stage, margin status, and treatment modality were all associated with overall survival (Table 2). Patients with CDCI of 1 (HR: 2.18, 95% CI: 1.30‐3.68; *P* = 0.003), positive surgical margins (HR: 3.37, 95% CI: 1.83‐6.24; *P* < 0.001), and T stage T2 (HR: 2.79, 95% CI: 1.24‐6.27; *P* = 0.01), T3 (HR: 4.87, 95% CI: 2.14‐11.09; *P* < 0.001), or T4 (HR: 6.70, 95% CI: 2.62‐17.10; *P* < 0.001) all were associated with worse OS. Treatment with trimodality therapy (surgery, CT, and RT) (HR: 0.45, 95% CI: 0.27‐0.78; *P* = 0.004) or surgery plus chemotherapy (HR: 0.54, 95% CI: 0.33‐0.90; *P* = 0.02) was associated with improved OS. In the small subset of patients (n = 26 patients, 7.9%) treated with only surgery and RT, there was no significant difference in OS compared to surgery alone (*P* = 0.26). Nodal status and surgery type (mastectomy vs partial mastectomy) did not significantly influence OS in our analyses. Multivariable logistic regression revealed only younger patients had higher likelihood of receiving trimodality therapy, while CDCI, race, year of diagnosis, facility type, insurance status, T or N stage, or margin status did not (Table 3).

**Table 2:** Univariate and multivariable Cox proportional hazards model for overall survival

	Univariate		Multivariable	
	HR (95% CI)	*P* value	HR (95% CI)	*P* value
Age	1.02 (1.01‐1.04)	<0.001	1.00 (0.98‐1.02)	0.92
Race				
White	Reference			
Nonwhite	1.10 (0.70‐1.72)	0.67	0.62 (0.35‐1.09)	0.10
Year of diagnosis	1.02 (0.94‐1.10)	0.63	1.01 (0.92‐1.11)	0.88
CDCI				
0	Reference			
1	2.83 (1.83‐4.38)	<0.001	2.18 (1.30‐3.68)	0.003
2	3.39 (1.56‐7.38)	0.002	2.41 (0.94‐6.20)	0.07
Facility program type				
Academic	Reference			
Non‐academic	1.03 (0.67‐1.59)	0.91	0.81 (0.49‐1.32)	0.39
Insurance status				
Private	Reference			
Government	2.13 (1.44‐3.16)	<0.001	2.07 (1.39‐3.32)	0.002
Uninsured	1.66 (0.70‐3.92)	0.25	3.00 (1.20‐7.48)	0.019
Clinical T stage				
T1	Reference			
T2	2.03 (1.08‐3.83)	0.03	2.79 (1.24‐6.27)	0.01
T3	4.20 (2.21‐7.99)	<0.001	4.87 (2.14‐11.09)	<0.001
T4	4.22 (2.00‐8.94)	<0.001	6.70 (2.62‐17.10)	<0.001
Clinical N stage				
N0	Reference			
N1	1.12 (0.70‐1.80)	0.63	1.04 (0.58‐1.88)	0.90
N2	2.72 (1.32‐5.63)	0.007	1.05 (0.44‐2.54)	0.91
N3	1.45 (0.20‐10.47)	0.71	1.37 (0.16‐11.50)	0.77
Surgery				
Mastectomy	Reference			
Partial mastectomy	0.78 (0.52‐1.17)	0.23	1.05 (0.62‐1.78)	0.86
Margin status				
Negative	Reference			
Positive	2.21 (1.28‐3.82)	0.004	3.37 (1.83‐6.24)	<0.001
Treatment modality				
Surgery alone	Reference			
Surgery +CT	0.42 (0.27‐0.67)	<0.001	0.54 (0.33‐0.90)	0.02
Surgery +RT	0.53 (0.26‐1.05)	0.07	0.66 (0.33‐1.35)	0.26
Surgery +CT + RT	0.30 (0.19‐0.49)	<0.001	0.45 (0.27‐0.78)	0.004

**Table 3:** Multivariable logistic regression for factors predictive of trimodality therapy

Characteristic	OR (95% CI)	*P* value
Age	0.95 (0.93‐0.97)	<0.001
Race		
White	Reference	
Nonwhite	1.01 (0.49‐2.08)	0.98
Year of diagnosis	1.09 (0.98‐1.21)	0.10
CDCI		
0	Reference	
1	0.94 (0.22‐4.04)	0.93
2	0.51 (0.10‐2.59)	0.42
Facility program type		
Academic	Reference	
Non‐academic	1.10 (0.58‐2.07)	0.78
Insurance status		
Private	Reference	
Government	1.88 (0.55‐6.50)	0.31
Uninsured	1.17 (0.31‐4.42)	0.82
Clinical T stage		
T1	Reference	
T2	1.49 (0.48‐4.64)	0.50
T3	1.19 (0.42‐3.36)	0.75
T4	1.99 (0.68‐5.85)	0.21
Clinical N stage		
N0	Reference	
N1	0.48 (0.14‐1.67)	0.25
N2/N3	0.64 (0.16‐2.52)	0.52
Margin status		
Negative	Reference	
Positive	3.62 (0.98‐13.33)	0.053

CDCI, Charlson/Deyo Comorbidity Index.

## 4 DISCUSSION

Carcinosarcoma is an aggressive subtype of breast cancer for which data on prognosis and ideal management is sparse. In this study, we used the NCDB to evaluate contemporary treatment approaches and their impact on survival in patients diagnosed with carcinosarcoma of the breast. Our study found that treatment with trimodality therapy or surgery and chemotherapy was associated with a significant improvement in overall survival. In addition, we identified other patient and treatment‐related factors associated with improved survival in this cohort.

Highlighting the underlying heterogeneity of various subtypes of metaplastic breast cancer, the most recent WHO guidelines have emphasized the relevance of a descriptive sub‐classification of these tumors given that carcinosarcoma, also known as metaplastic carcinoma with mesenchymal differentiation, differs not only histologically, but clinically from other metaplastic tumors.^4^ Due to the rarity of this diagnosis, evaluating practice patterns and outcomes on a national level are crucial to outlining optimal management. Here, we present a large study reporting patterns of care and survival outcomes of carcinosarcoma of the breast. Recent studies have shown that metaplastic breast cancer (MBC) is more aggressive than triple‐negative breast cancer (TNBC), an entity with which it has often been conflated. Compared to TNBC, patients diagnosed with MBC more likely present with advanced stage, have twice the rate of local recurrence, and more often die of their disease.^10^ Population‐based studies of MBC as a whole have shown worse overall survival than non‐MBC patients irrespective of hormone receptor status, implying MBC does independently confer a survival detriment as previously thought.^12^ Despite worse outcomes compared to non‐MBC breast cancers, including TNBC, no guidelines exist regarding the optimal treatment of these patients.

Most patients in our study were treated with mastectomy, perhaps due to the fact that only 20.6% of patients presented with T1 disease in our series and the majority of patients presented with large primaries. Mastectomy has been shown to be used more frequently in MBC compared to non‐MBC due to larger size at presentation, and rates are similar when adjusted for T stage.^13^ Despite this, patients who underwent breast‐conserving surgery did not experience inferior OS compared to mastectomy, consistent with well‐established randomized data.^14^ However, positive surgical margins were associated with worse outcomes in our series, consistent with the non‐MBC literature.^15^ While adjuvant therapy may reduce recurrence risks in the setting of a positive margin, it has not been shown to completely mitigate the worse outcomes seen in these patients.^16^ Unfortunately, the rates of re‐resection are not documented in the NCDB, and margins are only documented as negative vs positive, so the impact of close or focally positive margins on outcomes cannot be determined from the current analysis. Furthermore, only 22.5% of patients had clinically node‐positive disease at diagnosis, which is consistent with previous observations that carcinosarcoma has a propensity for local and hematogenous spread, rather than lymphatic spread.^17^ These patterns of failure inherent to carcinosarcoma may additionally explain why nodal involvement at diagnosis did not affect OS in our study. Given that disease‐specific mortality is primarily driven by high rates of distant metastases inherent to the natural history of carcinosarcoma, irrespective of nodal status, may at least partially explain why clinical node‐positivity did not impact survival in our series.

Metaplastic breast cancer is thought to be resistant to conventional chemotherapy with rates of progression as high as 83% in patients treated with neoadjuvant chemotherapy, and modest partial response to taxane‐based regimens and no responders to anthracycline and cyclophosphamide‐based regimens.^18^ Although details regarding specific chemotherapeutic agents and their doses are unavailable in our cohort, we did observe a substantial survival benefit to the use of chemotherapy in the current study, with 5‐year OS 70% in patients receiving systemic therapy compared to 41% in patients not treated with systemic therapy. A small subset of patients had hormone receptor‐positive disease in our series, and <5% of patients received adjuvant hormonal therapy, although receptor status has previously been shown to not affect prognosis in MBC.^19^ Data on the role of endocrine therapy are currently sparse, limited to isolated case reports.^20^ Of increasing interest in carcinosarcoma and MBC as a whole is the use of targeted therapies, particularly the epidermal growth factor receptor (EGFR) pathway, given that 70%‐80% of MBC overexpresses EGFR but not human epidermal growth factor receptor (HER)2/neu (c‐erbB2), for which no data currently exist on the effectiveness of targeted agents.^21^ Other identified pathways harboring potential targets in carcinosarcoma include the phosphoinositide 3‐kinase (PI3K)/AKT, MAP kinase signaling, and epithelial‐mesenchymal transition pathways.^22^

Interestingly, post‐lumpectomy radiation therapy was utilized in 71% of patients receiving lumpectomy, considerably lower than approximately 87% in non‐metaplastic, non‐carcinosarcoma breast cancer observed in other NCDB‐based analyses of breast cancer.^12^ Previously, population‐based studies have shown an independent improvement in both OS and disease‐specific survival advantage for patients receiving RT for patients with MBC when using the Surveillance, Epidemiology, and End Results (SEER) database.^23^ Adjuvant RT has been consistently shown to reduce breast cancer mortality both as a part of breast conservation therapy and in select postmastectomy patients.^24^, ^25^ The reasons for omitting RT as part of BCT in 29% of eligible patients, such as patient refusal or physician discretion, are unavailable in the NCDB and we are therefore unable to analyze these data in the current study.

Postmastectomy radiotherapy was utilized in 32% of carcinosarcoma patients treated with mastectomy, similar to approximately 30% of non‐carcinosarcoma mastectomy patients.^26^ All of the patients in our current series receiving PMRT had either pT3‐4, node‐positivity, or positive margins. Further conclusions in this cohort are difficult to draw from the current series, however, as the number of pathologic nodes nor RT nodal volume data was not consistently reported in the NCDB. No data exist currently to a role of PMRT in carcinosarcoma other than classic indications, and further investigation is warranted.

In our study, Kaplan‐Meier estimates did confirm treatment with RT was associated with a significant improvement in both 3‐ and 5‐year survival. On multivariable analysis, however, the small subset of patients who were treated with surgery and RT but not CT did not experience improved OS compared to surgery alone. This is unsurprising given that only 7.9% of patients were treated with surgery and RT, by far representing the smallest treatment group in our current series.

We found that patients treated with either trimodality therapy or surgery and chemotherapy experienced the greatest overall survival in this cohort. To determine factors associated with receipt of trimodality therapy, we performed logistic regression, with only younger age being significantly predictive. Notably, CDCI, T and N stage, as well as margin status did not predict for treatment with trimodality therapy. Nevertheless, trimodality therapy remained independently associated with improved overall survival on multivariable analysis. While the literature characterizing outcomes in metaplastic breast cancer as a whole is scarce, the characteristics and outcomes in carcinosarcoma are even less reported on. Data are often limited individual case reports or small case series with conflicting results about prognosis and response to treatment.^1^, ^27^, ^28^ In this series of carcinosarcoma of the breast, we demonstrate the previously unreported benefit of trimodality therapy in this patient population.

Although the strengths of our retrospective study include the large number of patients, there are considerable limitations. Our study is affected by limitations inherent to retrospective reviews and the nature of the NCDB as our data source. Carcinosarcoma histology is reported by individual institutions to the NCDB, but there is no central pathology review. Whether these patients had tumors with purely carcinosarcoma or had a partial component of carcinosarcoma is not reported, which may influence natural history and outcomes. There is potential selection bias as well, which may in part explain why patients treated with multimodal therapies experience improved OS. Patient selection may also explain why median survival of generally healthier patients, those with CDCI of 0, did not meet median overall survival, although CDCI did not predict for trimodality therapy. Further details of treatment, such as type and frequency of systemic therapy, radiotherapy modality, surgical management of the axilla, a standardized definition of positive margins, and use of salvage therapies are unavailable in the NCDB. Details on regional nodal irradiation volumes were inconsistently recorded and therefore excluded from our analysis. The NCDB records overall survival, but additional important endpoints including toxicity and quality of life data, local and regional control, as well as disease‐specific survival are omitted from the NCDB and therefore, we are unable to draw any conclusions with respect to these topics. Regardless, our work represents the largest series of this rare, aggressive disease to‐date and provides a basis for further prospective studies.

## 5 CONCLUSIONS

Carcinosarcoma of the breast is a rare, distinct entity of breast cancer characterized by highly aggressive behavior with a propensity for local and distant relapse despite low rates of lymphatic spread. Given a scarcity of studies investigating natural history and treatment outcomes in these patients, guidance on optimal management of these patients is severely lacking. Based on our findings, multimodal therapy provides a marked survival advantage, with patients treated with trimodality therapy consisting of surgery, chemotherapy, and radiotherapy experiencing the most favorable survival.

## CONFLICT OF INTEREST

We have no conflict of interest to disclose for this work.
